# Primary diffuse malignant epithelioid peritoneal mesothelioma of the greater omentum in an asbestos-naive patient: a case report

**DOI:** 10.3389/fmed.2026.1808853

**Published:** 2026-03-26

**Authors:** Xuemei Gan, Yinglan Tuo, Lan Huang, Qin Yang, Hong Liu

**Affiliations:** 1 Department of Hepatology, Dazhou Central Hospital, Dazhou, China; 2 Department of Pathology, Dazhou Central Hospital, Dazhou, China; 3 Department of Hepatobiliary Surgery, Dazhou Central Hospital, Dazhou, China

**Keywords:** case report, epithelioid mesothelioma, greater omentum, immunohistochemistry, malignant mesothelioma

## Abstract

**Background:**

Malignant mesothelioma is a rare malignant neoplasm of mesothelial origin, with primary involvement of the greater omentum being exceptionally uncommon. Nonspecific clinical manifestations often lead to misdiagnosis, and cases without asbestos exposure further increase diagnostic complexity.

**Case presentation:**

A 71-year-old male without asbestos exposure was admitted for recurrent diarrhea (>1 year), abdominal distension (>10 days), and unintentional weight loss (5 kg in 3 months). Physical examination revealed a 20-cm firm abdominal mass, and abdominal contrast-enhanced CT showed omental-peritoneal “cake-like” thickening with massive peritoneal effusion. Laparoscopic palliative tumor resection and peritoneal effusion drainage were performed, and postoperative pathology/immunohistochemistry (CK+, CR+, **CK5/6+, D2-40+, WT1+**, CD20-, CDX2-) confirmed epithelioid mesothelioma of the omentum. Postoperative management included anti-infective therapy, nutritional support, and Cinobufacini Capsule-based anti-tumor treatment, with stable disease control at 3-month follow-up.

**Conclusion:**

Definitive diagnosis of primary greater omental malignant mesothelioma relies on pathology and immunohistochemistry. For unresectable cases, palliative surgery combined with individualized adjuvant therapy may achieve favorable short-term outcomes, highlighting the need for early clinical suspicion in patients with unexplained abdominal symptoms.

## Introduction

Malignant mesothelioma is a rare mesothelial-derived malignancy, most commonly affecting the pleura or peritoneum (1). Primary involvement of the greater omentum is exceptionally rare, with only scattered clinical reports (2, 3). Its nonspecific manifestations (abdominal distension, palpable masses, weight loss) often lead to misdiagnosis as inflammatory lesions or metastatic carcinoma, delaying treatment. The etiology remains incompletely understood. Asbestos exposure is the primary risk factor, but 10%−20% of cases occur in asbestos-naive patients, suggesting contributions from chronic inflammation, viral infections, or genetic factors (4). With high malignancy and invasiveness, the disease carries a poor prognosis, with a median overall survival of merely 9–12 months (5). Current multimodal therapy recommends cytoreductive surgery (CRS) plus hyperthermic intraperitoneal chemotherapy (HIPEC) for resectable cases, and pemetrexed plus cisplatin as first-line chemotherapy for advanced or unresectable disease (6, 7). However, clinical data on primary greater omental malignant mesothelioma are scarce, and optimal individualized treatment strategies remain undefined.

Herein, we report a case of primary diffuse epithelioid mesothelioma of the greater omentum in an asbestos-naive patient, detailing its clinical features, diagnostic process and therapeutic course. By combining this case with literature review, we discuss the key diagnostic and therapeutic considerations, aiming to provide practical references for the early identification and personalized management of such rare cases.

## Case presentation

A 71-year-old male presented to our hospital on November 10, 2025, with a one-year history of recurrent diarrhea and a 10-day history of progressive abdominal distension. He experienced 3–4 episodes of yellow, loose stools daily without mucus or blood, which had not been systematically evaluated or treated. In the 10 days before admission, he developed worsening abdominal distension accompanied by anorexia. He denied abdominal pain, nausea, vomiting, fever, or night sweats. An unintentional weight loss of 5 kg was reported over the preceding three months. His past medical history included hypertension for over four years, managed irregularly without documented blood pressure control. He had a 40-pack-year smoking history (quit 1 year prior) and denied alcohol. His father died of tuberculosis. Physical examination revealed mild abdominal distension. A firm, cake-like mass approximately 20 cm in diameter was palpated in the mid-abdomen, with ill-defined margins and limited mobility. The abdomen was non-tender without rebound tenderness. The liver and spleen were not palpable. Shifting dullness was positive; bowel sounds were normal, and no lower extremity edema was present.

Laboratory tests showed the following abnormalities: hemoglobin 109g/L (reference: 120–160), serum albumin 32.94 g/L (40–55), prealbumin 158.2 mg/L (200–430), creatine kinase 31 U/L (50–310), C-reactive protein 52.6 mg/L (0–6), and lactic acid 2.33 mmol/L (0.6–2.2). Renal and thyroid function, electrolytes, lipid profile, coagulation tests, tumor markers, and routine stool analysis were unremarkable. Autoantibody testing revealed a positive antinuclear antibody (ANA) at a titer of 1:100 (granular pattern). Both purified protein derivative (PPD) skin test and T-SPOT. TB were positive. Urinalysis indicated microscopic hematuria (red blood cells 61/μl, reference: 0–17). Hepatitis B serology was positive for surface antibody (HBsAb) and core antibody (HBcAb); hepatitis C, syphilis, and HIV antibodies were negative.

**Figure 1:**
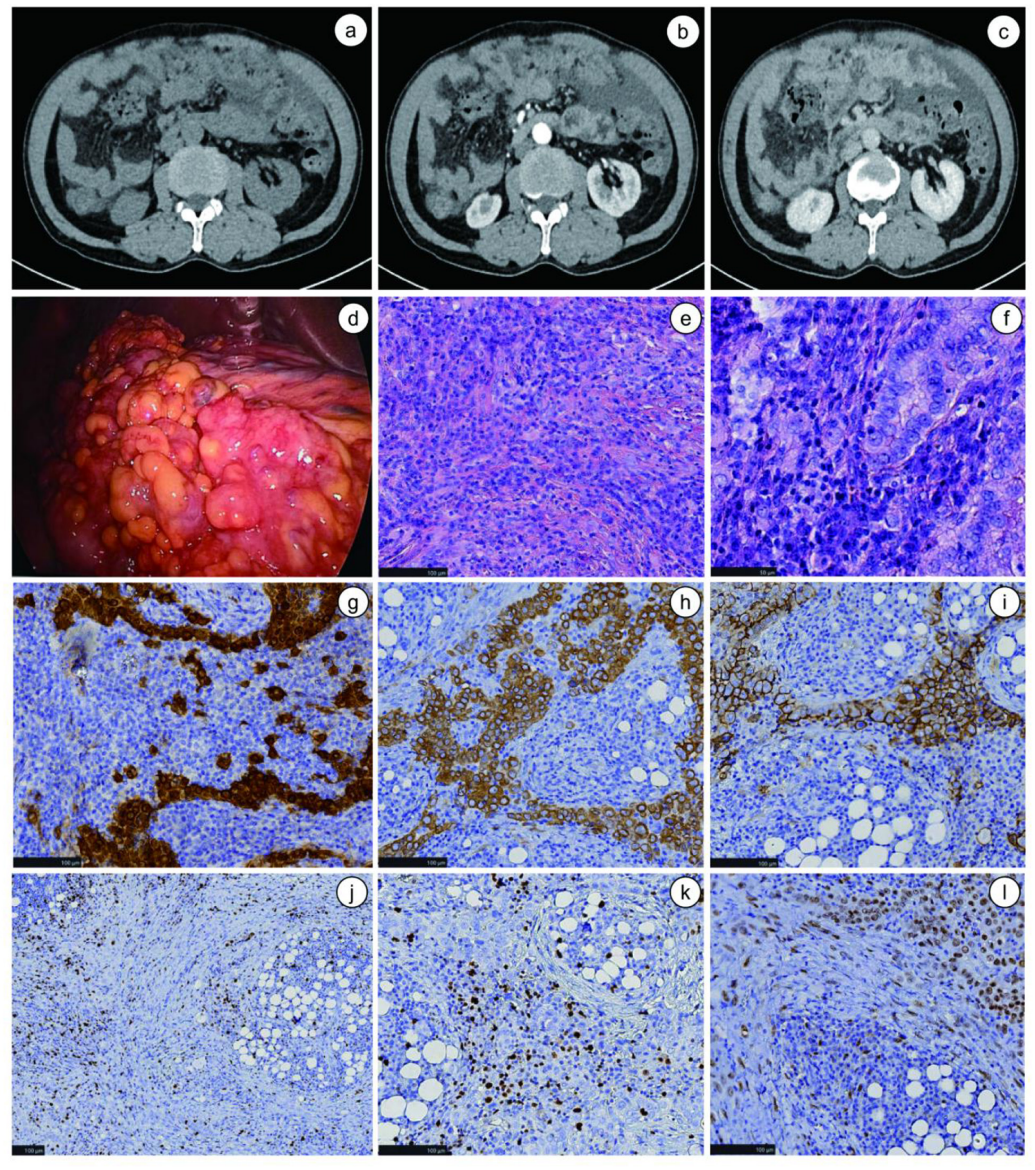
Preoperative CT findings, intraoperative tumor characteristics and postoperative pathological examination results. **(a–c)** Preoperative CT scans; **(a)** Plain scan phase; **(b)** Arterial phase; **(c)** Equilibrium phase; **(d)** Intraoperative location and distribution of the tumor; **(e)** The tumor shows irregular sheet-like and clustered distribution in the greater omentum, with partial glandular duct-like structures visible (HE staining, × 200); **(f)** Histologically, the tumor exhibits a predominantly tubular architecture, with minor components of cords, small clusters, and papillary structures. Cytologically, the tumor cells are mostly cuboidal, with slightly eosinophilic cytoplasm, fine chromatin, and visible small nucleoli. The stroma shows prominent desmoplasia, accompanied by abundant infiltration of lymphocytes and plasma cells (HE staining, × 400); **(g)** Calretinin positivity (IHC staining, × 200); **(h)** CK5/6 positivity (IHC staining, × 200); **(i)** D2-40 positivity (IHC staining, × 200); **(j)** Ki-67 expression is uniformly low across different regions of the tumor tissue. (IHC staining, × 100); **(k)** Ki-67 labeling index in hotspot areas is approximately 10% (IHC staining, × 200); **(l)** WT1 positivity (IHC staining, × 200).

Imaging studies included chest CT, which demonstrated bilateral emphysema and chronic infectious lesions. Transthoracic echocardiography revealed atrial enlargement and reduced left ventricular diastolic function. Cranial CT showed lacunar infarcts in the bilateral basal ganglia. Abdominal contrast-enhanced CT revealed nodular, cake-like thickening and marked enhancement of the greater omentum, peritoneum, and mesentery, along with mild wall thickening and enhancement of several small intestinal loops in the upper and mid-abdomen (Figures 1a–c), and a large volume of peritoneal effusion. Endoscopy showed chronic atrophic gastritis (C1, inactive); colonoscopy was normal.

On November 20, the patient underwent laparoscopic biopsy of the greater omental mass. Approximately 2,500 ml of turbid pale-yellow ascitic fluid was drained intraoperatively. The greater omentum was fused into a poorly demarcated mass adherent to the transverse colon, with scattered gray-white nodules on the parietal peritoneum (Figure 1d). Intraoperative frozen section suggested malignancy, favoring adenocarcinoma, prompting palliative tumor resection and drainage of ascites (Figures 1e, f). Final histopathology of the paraffin-embedded specimen confirmed a diagnosis of epithelioid mesothelioma of the omentum. Immunohistochemistry results were as follows: CK (+), Vim (+), CK7 (+), CR (+), CK5/6 (+), D2-40(+), WT1 (+), CK20 (−), CDX2 (−), Hepa (−), PAX-8 (−), and NKX3.1 (−) (Figures 1g–l).

Postoperatively, the patient received anti-infective therapy, fluid resuscitation, nutritional support, and anti-tumor treatment with Cinobufacini Capsules. Recovery was uneventful: the abdominal drain was removed on postoperative day 4, and the patient was discharged on day 8. He has continued regular oral Cinobufacini Capsules and remained under close follow-up. At the latest assessment on March 4, 2026 (three months post-discharge), he reported no recurrence of diarrhea or abdominal distension, with no abdominal mass on examination. Abdominal ultrasound showed no re-accumulation of ascites, and laboratory parameters (including hemoglobin, albumin, and C-reactive protein) had normalized, indicating stable disease control without treatment-related adverse events. Timeline of Diagnosis and Treatment (Table 1).

**Table 1:** Timeline of diagnosis and treatment.

**Time point**	**Key events**	**Examination & outcome**
Nov 10, 2025	Hospital admission for recurrent diarrhea and abdominal distension	Physical/laboratory evaluation: hypoalbuminemia, elevated D-dimer, and elevated C-reactive protein
Nov 13, 2025	Abdominal contrast-enhanced CT; colonoscopy	CT showed omental cake-like changes and peritoneal effusion; gastroscopy indicated chronic atrophic gastritis; colonoscopy was unremarkable
Nov 20, 2025	Laparoscopic greater omental biopsy; palliative tumor resection; peritoneal effusion drainage	Intraoperative findings: 2500 mL peritoneal effusion, omental nodules; frozen section: poorly differentiated adenocarcinoma
Nov 24, 2025	Postoperative day 4	Abdominal drainage tube removed; no obvious discomfort, stable vital signs
Nov 25, 2025	Postoperative pathology and immunohistochemistry results available	Confirmed epithelioid mesothelioma of the omentum (**CR+, D2-40+, CK5/6+, WT1+, CK+, Vim+, CK7+, CK20-, CDX2-, etc**.)
Dec 28, 2025	Postoperative day 8	Discharged smoothly; advised to take Cinobufacini Capsules regularly and attend follow-up
**Mar 4, 2026**	Regular follow-up (3 months after discharge)	No recurrence of diarrhea or abdominal distension; abdominal ultrasound showed no peritoneal effusion reaccumulation; laboratory indicators returned to normal

## Discussion

Malignant mesothelioma (**MM**) is a rare and aggressive neoplasm arising from mesothelial cells lining the serosal cavities, most commonly the pleura, followed by the peritoneum. Primary peritoneal mesothelioma (PPM) accounts for approximately 10%−15% of all MM cases, and its occurrence in the greater omentum as the dominant site is exceedingly rare, especially in patients without a history of asbestos exposure (6). The present case describes a 71-year-old asbestos-naive patient who presented with diarrhea and and abdominal distension and was ultimately diagnosed with primary diffuse malignant epithelioid peritoneal mesothelioma predominantly involving the greater omentum.

This case is unique due to its primary omental involvement in an asbestos-naive patient, challenging the conventional etiological association and broadening the clinical spectrum of peritoneal mesothelioma. While asbestos is the principal risk factor for MM, up to 20% of patients report no known exposure, suggesting alternative or spontaneous pathogenetic mechanisms (8). Our patient's occupational and environmental history was thoroughly reviewed and revealed no asbestos contact, reinforcing the notion that peritoneal mesothelioma can occur in asbestos-naive individuals.

The diagnostic workup of this patient illustrates the typical challenges encountered in identifying primary omental mesothelioma. Preoperative contrast-enhanced computed tomography (CT) of the abdomen and pelvis revealed a large, heterogeneous omental mass (approximately 20 cm) with moderate enhancement and no significant ascites or diffuse peritoneal thickening (Figure 1). Based on these imaging findings, the differential diagnosis included primary omental tumor, peritoneal carcinomatosis, and rarely, tuberculous peritonitis. The patient underwent exploratory laparoscopy with omental biopsy. Intraoperatively, the tumor was found to be predominantly confined to the greater omentum, with only a few scattered small (< 5 mm) nodules on the parietal peritoneum; there was no evidence of diffuse peritoneal thickening, widespread carcinomatosis, or ascites. Comprehensive exploration of the abdominal cavity, including inspection of the liver, spleen, ovaries, and gastrointestinal serosa, revealed no other primary tumor or metastatic deposits. These intraoperative findings were crucial in supporting a primary omental origin rather than diffuse peritoneal mesothelioma or metastatic disease.

Histopathological examination of the resected specimen showed an epithelioid neoplasm arranged in tubulopapillary and solid nested patterns. Histopathologically, the tumor displayed a predominantly tubular architecture with focal tubulopapillary and solid nested patterns, consistent with the epithelioid subtype of mesothelioma. The accompanying prominent desmoplasia and lymphoplasmacytic infiltration suggested a host immune response, which may have prognostic implications. Immunohistochemically, the tumor cells were diffusely and strongly positive for Calretinin, CK7, and vimentin, while negative for CK20 and PAX8. Additional mesothelial markers, including WT1 (nuclear positivity) and D2-40 (membranous positivity), were also performed and confirmed the mesothelial lineage. The Ki-67 proliferation index was approximately 10%. The immunohistochemical profile effectively excluded metastatic carcinoma (CK20–, PAX8–) and other peritoneal tumors.

Differential diagnosis of omental tumors is broad and includes metastatic adenocarcinoma (most commonly from the gastrointestinal tract or ovary), primary peritoneal serous carcinoma, sarcomatoid carcinoma, lymphoma, and tuberculous peritonitis. In our case, the negativity for CK20, CDX2, and PAX8 effectively ruled out a colorectal, pancreatobiliary, or ovarian origin. The positive staining for mesothelial markers (Calretinin, WT1, D2-40) and the absence of a primary tumor elsewhere on imaging and intraoperative exploration strongly supported malignant mesothelioma over a metastatic process. Peritoneal carcinomatosis from an unknown primary was excluded by the lack of a primary tumor on imaging and the IHC profile. Tuberculous peritonitis was considered, particularly because the patient had a positive T-SPOT result. However, tuberculous peritonitis was ruled out based on the absence of clinical symptoms (fever, night sweats, weight loss), intraoperative findings (no tubercles, caseous necrosis, or adhesions typical of TB peritonitis), histopathology (no granulomas, caseous necrosis, or acid-fast bacilli on Ziehl-Neelsen staining), and ascites fluid analysis (low adenosine deaminase level). Other peritoneal tumors, such as leiomyosarcoma and lymphoma, were excluded by morphology and negative immunostaining for desmin, LCA, and S100. The diagnosis of epithelioid mesothelioma was thus firmly established. This histological subtype, which carries prognostic significance, warrants further discussion.

Histologically, malignant mesothelioma is categorized into epithelioid, sarcomatoid, and biphasic subtypes, with the epithelioid variant—as seen in our patient—being the most common and associated with a relatively better prognosis (9). The nonspecific clinical presentation (abdominal distension, weight loss, ascites) and the low diagnostic yield of ascitic cytology often lead to initial misdiagnosis. In this context, immunohistochemistry is indispensable for definitive diagnosis. Our IHC panel (CK+, Vim+, CR+, CK5/6, D2-40, WT1, CK20–, CDX2–) effectively excluded gastrointestinal carcinomas and confirmed epithelioid mesothelioma. Therefore, in middle-aged and elderly patients with unexplained diffuse peritoneal thickening and ascites, malignant mesothelioma should remain a key differential diagnosis even without asbestos exposure, and prompt laparoscopic biopsy is warranted.

Comparison with similar cases in the literature reinforces the rarity and diagnostic complexity of primary omental mesothelioma. Cravero et al. recently reported a case of primary peritoneal mesothelioma affecting the greater omentum that initially mimicked omental infarction (10). Similar to our patient, their case occurred in an asbestos-naive individual and required immunohistochemistry for definitive diagnosis. However, our case differs in the IHC panel (including CK5/6, WT1 and D2-40), contributing additional data to the growing literature. Mehta et al. described a primary sarcomatoid hepatic mesothelioma and provided a literature synthesis of rare intra-abdominal variants, emphasizing the need for a high index of suspicion (11). Betancourt et al. reported an epithelioid primary peritoneal mesothelioma with nonspecific abdominal presentation, highlighting diagnostic challenges (12). Frontario et al. presented a case of primary peritoneal mesothelioma presenting as small bowel obstruction and reviewed the diagnostic approach (13). Collectively, these reports underscore the diverse clinical and pathological manifestations of extrapleural mesothelioma and the importance of a comprehensive diagnostic workup. Unlike the case reported by Cravero et al., which mimicked omental infarction, our patient presented with chronic diarrhea and progressive abdominal distension, further expanding the clinical spectrum of this rare entity. Moreover, our comprehensive IHC panel (including CK5/6, WT1, and D2-40) provides additional diagnostic confirmation not reported in some earlier cases.

Treatment and short-term follow-up: the patient underwent laparoscopic partial resection of the greater omental tumor followed by adjuvant cinobufacini therapy. At the 3-month follow-up, the patient remained alive with no clinical or imaging evidence of disease progression. However, this short follow-up period is insufficient to assess long-term oncologic outcomes, and no objective response criteria (e.g., RECIST) were applied.

This study has several limitations. First, the follow-up duration is only three months, precluding any meaningful assessment of long-term survival or recurrence. Second, the patient did not receive standardized systemic therapy according to a clinical trial protocol, and no objective tumor response assessment was performed. Third, due to limited tissue availability, certain immunohistochemical markers with potential prognostic value, such as BAP1 and MTAP, were not evaluated. Fourth, as a single case report, the findings may not be generalizable to all patients with peritoneal mesothelioma. Despite these limitations, our report adds to the scarce literature on primary omental mesothelioma in asbestos-naive patients and emphasizes the diagnostic approach.

In conclusion, we present a rare case of primary diffuse malignant epithelioid peritoneal mesothelioma involving the greater omentum in an asbestos-naive patient. The diagnosis was established through a combination of intraoperative findings, histomorphology, and a comprehensive immunohistochemical panel that excluded metastatic carcinoma and other peritoneal tumors. This case highlights the importance of considering mesothelioma in the differential diagnosis of omental masses, even in the absence of asbestos exposure, and underscores the need for a structured diagnostic framework. Longer follow-up and accumulation of similar cases will help refine our understanding of the clinical behavior and optimal management of this rare entity.

### Patient perspective

I have been troubled by recurrent diarrhea for more than a year, but I didn't pay much attention to it and didn't receive standardized treatment. More than 10 days ago, I started to have abdominal distension which got worse day by day, and I even lost my appetite and lost 5 kilograms in three months, which made me very anxious. After being admitted to the hospital, the doctors conducted a series of detailed examinations for me, from laboratory tests to imaging examinations, and finally confirmed the diagnosis through laparoscopic biopsy. The surgery went smoothly, and the postoperative care was excellent, with minimal pain. After discharge, I have been taking Cinobufacini Capsules as prescribed by the doctor and attending regular follow-up visits. Now, two months after the operation, my abdominal distension and diarrhea have completely disappeared, I can eat normally, and my physical strength has gradually recovered. I am very satisfied with this treatment effect. The personalized treatment plan not only controlled the disease but also had no obvious side effects. I hope my case can help other patients with similar symptoms get timely diagnosis and effective treatment, and not delay the best treatment time due to unclear diagnosis.

## Data Availability

The datasets presented in this article are not readily available because the datasets generated and analyzed during this study are not publicly available due to ethical restrictions and patient privacy concerns. The informed consent signed by the patient explicitly states that the clinical data and images will be used solely for this research publication and will not be shared publicly. However, de-identified data may be made available from the corresponding author upon reasonable request and with permission from the institutional ethics committee requests to access the datasets should be directed to Hong Liu, scliuhong123@163.com.
